# Does Feather Corticosterone Reflect Individual Quality or External Stress in Arctic-Nesting Migratory Birds?

**DOI:** 10.1371/journal.pone.0082644

**Published:** 2013-12-31

**Authors:** Pierre Legagneux, N. Jane Harms, Gilles Gauthier, Olivier Chastel, H. Grant Gilchrist, Gary Bortolotti, Joël Bêty, Catherine Soos

**Affiliations:** 1 Département de biologie & Centre d'études nordiques, Université du Québec à Rimouski, Québec, Canada; 2 Department of Veterinary Pathology, University of Saskatchewan, Saskatoon, Saskatchewan, Canada; 3 Département de biologie & Centre d'études nordiques, Pavillon Vachon, Université Laval Québec, Québec, Canada; 4 Centre d'Études Biologiques de Chizé, CNRS-UPR 1934, Carrefour de la Canauderie, Villiers-en-Bois, France; 5 Environment Canada, National Wildlife Research Centre, Ottawa, Ontario, Canada; 6 Department of Biology, University of Saskatchewan, Saskatoon, Saskatchewan, Canada; 7 Environment Canada, Saskatoon, Saskatchewan, Canada; 8 Department of Veterinary Pathology, University of Saskatchewan, Saskatoon, Saskatchewan, Canada; Arizona State University, United States of America

## Abstract

The effects of environmental perturbations or stressors on individual states can be carried over to subsequent life stages and ultimately affect survival and reproduction. The concentration of corticosterone (CORT) in feathers is an integrated measure of hypothalamic–pituitary–adrenal activity during the molting period, providing information on the total baseline and stress-induced CORT secreted during the period of feather growth. Common eiders and greater snow geese replace all flight feathers once a year during the pre-basic molt, which occurs following breeding. Thus, CORT contained in feathers of pre-breeding individuals sampled in spring reflects the total CORT secreted during the previous molting event, which may provide insight into the magnitude or extent of stress experienced during this time period. We used data from multiple recaptures to disentangle the contribution of individual quality *vs.* external factors (i.e., breeding investment or environmental conditions) on feather CORT in arctic-nesting waterfowl. Our results revealed no repeatability of feather CORT within individuals of either species. In common eiders, feather CORT was not affected by prior reproductive investment, nor by pre-breeding (spring) body condition prior to the molting period. Individual feather CORT greatly varied according to the year, and August-September temperatures explained most of the annual variation in feather CORT. Understanding mechanisms that affect energetic costs and stress responses during molting will require further studies either using long-term data or experiments. Although our study period encompassed only five years, it nonetheless provides evidence that CORT measured in feathers likely reflects responses to environmental conditions experienced by birds during molt, and could be used as a metric to study carry-over effects.

## Introduction

Hormones play an essential role in the study of life-history trade-offs because they link environmental condition and phenotypic expression [1]. Glucocorticoids are of particular interest when examining how individuals respond to environmental variation, because they are the main mediators of allostasis allowing an organism to cope with both predictable and unpredictable conditions [2]–[4]. Corticosterone (CORT), the primary glucocorticoid in birds, has been investigated in numerous studies attempting to identify physiological mechanisms linking individual state to environmental parameters and fitness consequences. Measurement of baseline CORT is difficult in wild populations because CORT levels measured in plasma and feces rise shortly after capture [5]. CORT measured in feathers are not affected by capture when a fully grown feather is collected, and incorporate the amplitude and duration of all CORT secreted during the period of feather growth, and thus represent an integrated measure of both baseline and stress-induced CORT secretion during the molt period [6]. This technique is relatively recent, and an understanding of the information conveyed by feather CORT in wild populations is only beginning to be unravelled [7]–[9].

Immediately following reproduction, migratory birds are in a critical and energetically demanding period when they must accumulate lean tissue and fat for migration and molt [10]–[12]. Understanding the mechanisms of carry-over effects of breeding effort on body condition and molt is logistically challenging in migratory species because molt often occurs on distinct locations away from breeding sites, which makes the follow up of individuals almost impossible [13], [14]. Some studies investigated, either empirically or experimentally, how reproductive activities could delay timing of molt [15]–[18] and potentially induce lean tissue mass reduction [19], lower insulation [20], or a decrease in feather quality [21]. Late breeders can adjust their timing by molting their feathers more rapidly than early ones [18]. However, there are associated costs based on growing evidence showing that molt speed, and therefore, feather growth rate, influences the quality of ornaments, aerodynamics, and insulation [21]–[26]. Although less investigated, environmental conditions can also be important drivers of molting date [27] and determine feather quality [28].

**Figure 1:**
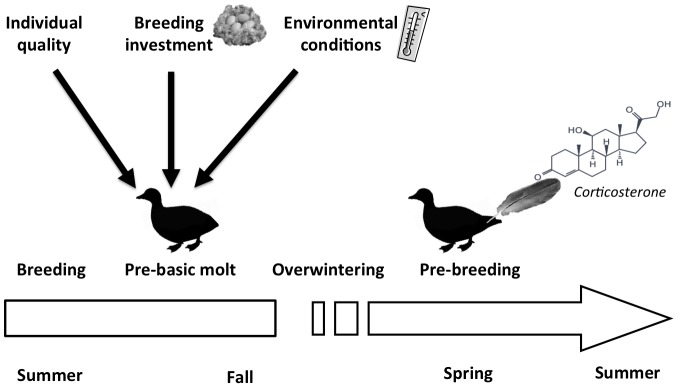
Time scale of partial capital breeders breeding in the High Arctic. From multiple recaptures, we tested whether individual quality, reproductive investment or environmental conditions (black arrows) explained variation in feather CORT from birds captured in spring during the pre-breeding period. The effects of reproductive investment or environmental conditions were investigated only in Common eiders.

In some bird species, like Anatidae, all flight feathers molt simultaneously once a year in late summer, after breeding. During this period, birds are flightless and thus potentially more vulnerable to predation, which can represent an additional source of stress compared to birds that molt their feathers sequentially. A feather collected in spring, prior to breeding, could thus provide an indication of the stress responses or energetic demands experienced by the molting birds approximately nine months earlier (fig. 1).

Thus, feather corticosterone in these species can potentially be a very useful tool for studying the role of carry over effects of conditions experienced during the previous molt, on reproduction and survival in the subsequent breeding period.

Using multiple recaptures of the same individuals, our aim was to disentangle the contribution of individual quality *vs.* external factors (fig. 1) on feather CORT in two wild bird species, the common eider *Somateria mollissima* and the greater snow goose *Chen caerulescens*. Although very valuable, intra-individual variation and repeatability in physiological traits are rarely investigated [29] and not yet reported for feather CORT. If individual quality, here viewed as an intrinsic (i.e. genetic) trait [30], is a strong determinant of feather CORT, we would expect a high temporal repeatability of relative feather CORT [31], [32].

Alternatively, if CORT in feathers was highly variable among years within the same individual, this would suggest that external factors such as breeding investment or environmental conditions have a stronger influence on CORT levels than intrinsic factors (e.g., individual quality). Secondly, in the common eider, we examined whether the timing of reproduction and breeding investment (breeders vs. non breeders), and pre-breeding mass affect CORT sequestered in feathers during molt immediately following reproduction. Finally, we investigated correlations between environmental conditions (weather conditions and trophic interactions) and feather CORT in common eiders.

## Materials and Methods

### Ethics Statement

This study adhered to guidelines of the Canadian Council on Animal Care, and all protocols were reviewed and approved by the University Committee on Animal Care and Supply - Animal Research Ethics Board of the University of Saskatchewan (Protocol Number 20100063 to CS), Environment Canada's Animal Care Committee (Protocol Numbers: EC-PN-07-008 (2007), EC-PN-08-026 to EC-PN-11-026 (2008 to 2011) to HGG) and by the Université du Québec à Rimouski (CPA-42-10-78 to JB).

### Study species and field methods

Geese and eiders are partial capital breeders that molt all their flight feathers simultaneously after the breeding season. During incubation, female eiders and geese lose up to 40% of their body mass due to fasting [33]. After hatching, female eiders must recover and regain body condition before initiating molt, another energetically costly event [34]. For this study we used data from long-term monitoring studies in which birds were fitted with permanent markers with unique combination to allow resightings in subsequent years [35], [36]. Captures occurred prior to breeding in spring staging areas for geese and on the breeding colony for eiders. Geese were captured from 2006 to 2009 in the Saint Lawrence estuary near Québec city [47°00′N 70°33′W, see details in 36]. Eiders were captured on Mitivik Island (64°02′N, 81°47′W) in the East Bay Migratory Bird Sanctuary, Southampton Island, Nunavut, Canada from 2007–2011 [37].

From late April to mid-May 2006–2009, we captured geese using baited canon-nets [38]. Adult females (>1 year old) were weighed and measured shortly after capture. Females were individually marked with neck collars [39]. From mid-June to early July, eiders were captured using large flight nets as eiders flew over the island. Eiders were weighed and measured shortly after capture. Adult female eiders were banded with a metal band and two coloured alphanumeric Darvic bands (Pro- Touch, Saskatoon, Canada) [40]. All female common eiders were also marked with a unique color and shape combination of two temporary plastic nasal markers (Juno Inc., Minneapolis, MN) so that nasal tagged individuals could be identified on nests. We attached nasal markers with synthetic absorbable suture monofilament (Polydiaxanone suture, 2.0 or 3.0 metric; Ethicon, Markham, Canada). At capture, one flight feather (a central undercovert in greater snow geese and the second right rectrix in common eider) was plucked from each individual. Feathers were stored in an envelope in a dark and dry place until measurements in the laboratory.

We first selected feathers from all recaptured individuals (N = 16 geese and N = 65 eiders) to assess individual repeatability. For common eiders, the effect of previous breeding investment was assessed for 29 recaptured individuals for whom we had previous breeding information. We determined if these females attempted to breed or not in the previous year, based on whether they were resighted on nest and, for those that bred, we determined laying date (N = 16) and nesting success (a nest was considered successful when at least one egg hatched, N = 15). Precise information on reproduction (laying date of the first egg and subsequent nest success) was available through the daily monitoring (via a maximum of seven blinds within the colony) and nest visitation (15 to 78 nests each year) of marked females. Laying date of females was determined by monitoring >358 nests annually at the colony.

We investigated possible links between relative pre-laying biomass for a given year and CORT levels sequestered in new feathers grown during the pre-basic molt of the same year immediately following breeding (though feathers were plucked in the following year). We restricted these analyses to birds captured and weighed during the pre-laying period in order to avoid any change in body mass due to egg laying. To do so and for each year, we only included data from birds caught before the date at which >2.5% of the population had started laying. Individuals with known laying dates were subsequently added to the dataset if known laying date was later than capture date (with a buffer of 3 days to account for potential error on laying date estimation). This procedure was applied during all the years of the study, which yielded a sample of 16 eiders with known laying dates and 17 with known body mass.

Finally, we investigated the effect of environmental covariates on feather CORT variation with a larger sample size (N = 652) that included all captured individuals from 2007 to 2011 for which we had feather CORT data. For this last analysis, we included body size as an individual covariate because structural body size is a static variable that remains similar over time once eiders reach sexual maturity. Body size was assessed using a principal component analysis on morphometric measurements (culmen, bill, tarsus and head lengths). The four variables had loadings ranging from 0.34 to 0.57 on the first axis (PC1), which explained 56.2% of total variation in the body size data. We used individual PC1 scores as a measure of body size.

### Environmental covariates

We addressed the influence of environmental conditions on CORT during molt only on eiders because not enough data was available in geese. Molting in females occurs outside the colony in our studied eider population, presumably in East Bay (Southampton Island) but precise information on molting period and molting sites are still lacking [41]. Based on our knowledge of the study system, we considered a two months molting period [42].

#### Climate

We used different climate indices (temperature, precipitation, and North Atlantic Oscillation). At the regional scale, large-scale indices are often better predictors of ecological processes than local climatic variables, likely because features of several weather components are combined [43]. The North Atlantic Oscillation (NAO) is a major source of atmospheric mass balance measured as the mean deviation in average sea level pressure between the subarctic and subtropical Atlantic [44]. By influencing the speed and direction of westerly surface winds across the North Atlantic, the NAO influences weather conditions over eastern Canada-USA and northern Europe [44], [45]. We obtained daily values of the NAO indices from the Climate Prediction Center of the National Weather Service (http://www.cpc.ncep.noaa.gov). We also used average values of temperature and precipitation from the meteorological station of Coral Harbour located 42 km from our study site and available at http://www.climat.meteo.gc.ca/climateData.

Finally, we used two indices of ocean primary production of East Bay from remote-sensing data. Satellite-derived measurements of surface chlorophyll (Chla) and sea surface temperature (SST) are indication of ocean primary production [46]. Chla and SST were extracted using a resolution of 9×9 km from MODIS satellite images (SST 4 micron night and Chla). We selected one pixel centered on the bay and extracted mean values values of SST and Chla from August and September of each year. Using a greater area (108×27 km) covering more potential moulting sites did not affect the results (not shown). Data were available at http://gdata1.sci.gsfc.nasa.gov/.

#### Trophic interactions

Ducklings are especially vulnerable to predation and females tending broods may increase their parental investment to defend their offspring when predation risk is high. Therefore, we expected that the level of duckling protecting behaviours displayed by females in response to predatory attacks could affect their ability to regain condition, and hence affect their feather CORT level during molt. The main predator of common eider eggs and ducklings on East Bay Island is the herring gull (*Larus argentatus*). As an index of gull abundance, we calculated the cumulative numbers of gulls counted daily around the island, including both breeders and transient birds. We considered the period 30 June (median hatching date) to 15 August (end of the colony monitoring), which was common to the five years of the present study. We assumed that gull abundance measured during that period was a good proxy for predation risk experienced by females during brood rearing.

### CORT assays

Feather CORT assays were performed at the University of Saskatchewan, Canada, and at the Centre d'Études Biologiques de Chizé, France, for eiders and geese respectively. We followed previously established protocols [6], [47]. In eiders, inter and intra-assay coefficients of variation (%CV) were 13.7 and 5.4% respectively (N = 18). In geese, such %CVs were 10.1 and 5.1% respectively (N = 16). The lowest detectable concentration was 10.3 or 11.4 pg CORT/assay tube (for University of Saskatchewan and CEBC respectively) while the lowest measurement was tenfold for both eiders and geese. The length of the feather was measured, the calamus was removed and discarded, and then the sample was cut into pieces less than 5 mm^2^ with scissors. We then added 10 ml of methanol (HPLC grade, Fisher Scientific, Fairlawn, NJ, USA) and placed the samples in a sonicating water bath at room temperature for 30 min, followed by incubation at 50°C overnight in a shaking water bath. The methanol was then separated from feather material by vacuum filtration, using a plug of synthetic polyester fibre in the filtration funnel. The methanol extract was placed in a 50°C water bath and subsequently evaporated in a fume hood. Extract residues were reconstituted in a small volume of phosphate-buffered saline (0.05 M, pH 7.6) and frozen at −20°C until analysed by radioimmunoassay (RIA). We assessed the efficiency of the methanol extraction by including feather samples spiked with a small amount (approximately 5000 CPM) of ^3^H-corticosterone in the extraction. RIA assays were performed on reconstituted methanol extracts, and samples were measured in duplicate. Serial dilutions of feather extracts produced displacement curves parallel to the standard curves. Data values are expressed as pg CORT per mm of feather, which gives a valid estimate of CORT per unit time of feather growth [6], [47].

### Statistical analyses

Intra-individual repeatability of feather CORT among years was calculated using the within and between-variance components in a linear mixed effects model, using the restricted maximum-likelihood method (REML) with bird identity as the grouping random factor [48], [49]. Data were analysed using R studio v2.1 (R Development Core Team) and the package rptR to calculate repeatability (R) and associated standard errors based on bootstraps (1000 iterations) that generated the distributions of likelihood ratios.

To examine variations in feather CORT, we used generalized linear mixed models with climatic and predation indices as explanatory covariates. Year was considered as a random factor to account for multiple samples per year. Our dataset contains yearly covariates (temperature, SST etc.) that represent a case of potential pseudo-replication. To avoid any pseudo-replication and over-parameterization (only 5 years studied), no more than one yearly parameter was entered in a competing model. Models were then ranked through a model selection procedure.

The model with the lowest AICc was chosen [50], unless the differences in AICc (ΔAICc) were smaller than 2, in which case the simplest model was selected [51]. All data were log-transformed to follow assumptions of normality. Maximum and restricted maximum likelihood fitted models were used for model comparison and parameter estimation, respectively.

## Results

### Repeatability

**Figure 2:**
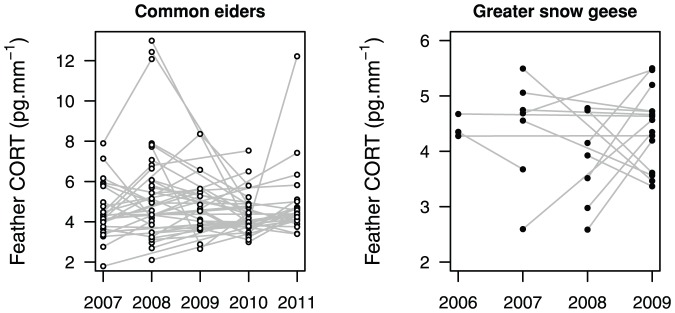
Repeatability of feather corticosterone. Repeatability is shown for the common eider (in white) and the greater snow geese (in black). Levels of feather CORT of recaptured individuals in different years are represented with lines connecting same individuals. (N = 65 and 16 respectively). Note that scale of the Y-axis differs between the two graphs.

The repeatability of feather CORT within individual eiders sampled more than once in different years was low and not significant (R = 0.1±0.09 SE; P = 0.76; N = 65; Fig. 2a). When examining only individuals recaptured in consecutive years, the repeatability was still very low and non-significant (R = 0.04±0.13 SE; P = 0.58; N = 29). The same results were found in geese, with a repeatability of almost nil (R = 0.01±0.15 SE; P = 0.50; N = 16 for all individuals and R = 0.01±0.13 SE; P = 0.29; N = 8 for individuals sampled during consecutive years; Fig. 2b).

We also considered repeatability analyses using relative feather CORT values (x-median of the yearly sample) for eiders, the species with the largest sample size. Similar results were found (R = 0.14; P = 0.45 and R = 0.31; P = 0.19 for all recaptures and for consecutive years respectively).

### Influence of breeding investment

**Figure 3:**
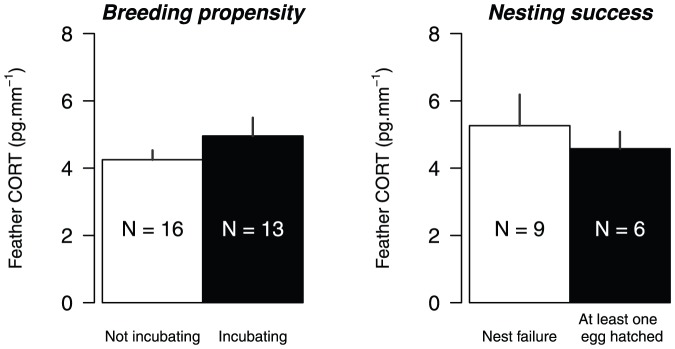
Feather corticosterone and previous breeding investment in common eiders. Average feather CORT (+S.E.) with information on breeding propensity or nesting success during the previous year at East Bay. The effect of breeding propensity or nesting success on feather CORT was non significant.

**Figure 4:**
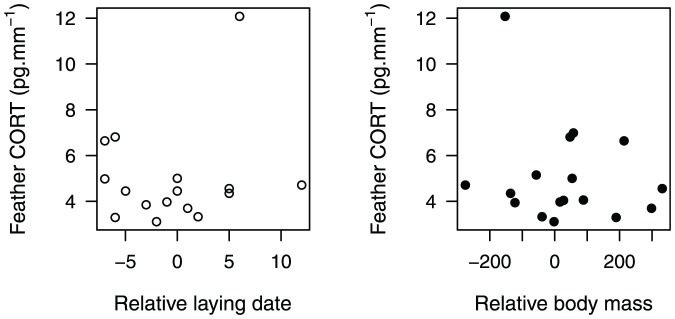
Feather corticosterone and previous laying date and body mass in common eiders. Relationship between feather CORT and previous laying date (relative to the median of all monitored individuals for a given year, white dots) and body mass measured in late June in female common eiders (residuals from a regression between capture date and mass for a given year, black dots). These graphs were performed from birds captured in consecutive years.

CORT in eider feathers was not related to previous breeding investment (breeding propensity or nesting success; F_1,27_ = 1.11; P = 0.30 and F_1,13_ = 0.18; P = 0.68 respectively; Fig. 3). Feather CORT in eiders was not related to relative pre-breeding body mass nor to relative laying date of the previous breeding season (F_1,15_ = 0.05; P = 0.82 and F_1,14_ = 0.50; P = 0.49 respectively, Fig. 4).

### Environmental conditions

**Figure 5:**
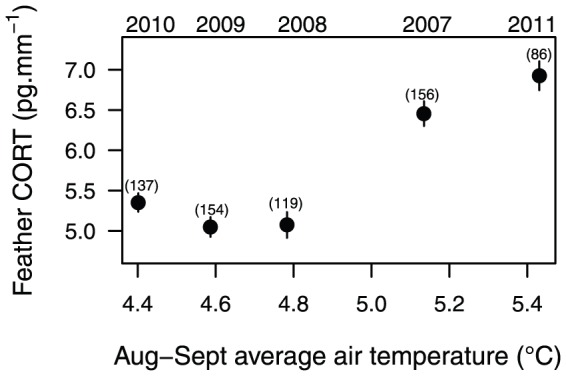
Feather corticosterone in common eiders and late summer air temperature. Average feather CORT (±SE) according to the average air temperature in August and September recorded in Coral Harbour (Southampton Island, Nunavut). Years of capture (above) and sample size (under brackets) are provided.

Our index of predation during the hatchling stage was not related to feather CORT in eiders (Table 1). Climatic variables such as Sea Surface Temperature or primary production (Chla) were poorly ranked (see model selection in Table 1). However, air temperature was the best variable explaining yearly variation in eider feather CORT (Table 1). Eider feather CORT levels were positively related to late summer temperatures (β = 0.085±0.017 SE Fig. 5) but not temperature variation (Table 1). A model with body size and temperature was also within 2 AICc (Table 1). The effect of body size was marginal here. Larger individuals tended to present lower CORT in their feathers (β = 0.011±0.008 SE).

**Table 1:** Model selection of the effects of local (average Aug–Sept temperature T and associated standard deviation TSD, precipitation PPT) and global (NAO) climate, and remote sensing covariates (SST and CHLa) on feather CORT variation on female common eiders.

Models	k	AICc	ΔAICc	ω_i_	LL
T	4	353.47	0	0.39	−172.7
T+Size	5	353.75	0.29	0.34	−171.83
TSD	4	357.32	3.85	0.06	−174.63
TSD+Size	5	358.09	4.62	0.04	−174
NAO	4	358.65	5.18	0.03	−175.29
Null	3	359.51	6.04	0.02	−176.74
NAO+Size	5	359.62	6.15	0.02	−174.76
CHLa	4	359.77	6.3	0.02	−175.85
Size	4	360.26	6.79	0.01	−176.1
PPT	4	360.35	6.88	0.01	−176.14
STT	4	360.38	6.91	0.01	−176.16
CHLa+Size	5	360.48	7.01	0.01	−175.19
HEGU	4	360.55	7.08	0.01	−176.24
PPT+Size	5	361.27	7.8	0.01	−175.59
HEGU+Size	5	361.35	7.88	0.01	−175.63

An index of predation pressure (number of gulls counted from June to mid august) was also considered. An index of eider body size (1st axis of a PCA based on head, culmen, wing and tarsus lengths) was also incorporated. Year was considered as a random factor. N observations = 652 females. k = number of parameters, ωi = model AICc cumulative weight, LL = log-likelihood.

## Discussion

To our knowledge, our study is the first to examine individual repeatability of feather CORT in wild birds. Feather CORT is an integrated measure of the stress responses and energetic demands experienced by an individual during the period of feather growth [6], [52]. In the literature, there are few examples of repeatability of CORT responses in birds. For instance, repeatability of stress-induced plasma CORT was observed in Adelie penguins (*Pygoscelis adeliae*) [53] and in female but not male Zebra finches (*Taeniopygia guttata*) [54]. Repeatability of fecal CORT levels was observed in a semi-captive population of geese (*Anser anser*) across several seasons [55]. Studies exploring intra-individual variability of baseline plasma CORT in wild animals have inconsistent results with repeatability being either context or sex-dependent [31], [54], [56]. Angelier et al. [32] reported high repeatability of basal CORT among individuals (especially in females) and an effect of reproductive investment on basal CORT measured at the beginning of reproduction in the wandering albatross (*Diomedea exulans*). In the present study, no repeatability in feather CORT level was found in either common eiders or snow geese, two species that share similar ecology and life history traits suggesting a prominent role of external conditions (such as previous breeding investment, climatic or foraging conditions). The timing of sampling also differs between our study (molting) and others (mostly pre-laying or breeding) focusing on CORT repeatability. Indeed, repeatability of baseline CORT levels can vary depending on stage of annual life cycle [56]. In free ranging tree swallows (*Tachycineta bicolor*), repeatability of plasma CORT was found within the breeding season, but not across seasons or years [31]. In captive house sparrow (*Passer domesticus*), basal CORT repeatability was lower during molt than at other stages of the life cycle [56]. Since, repeatability of basal CORT during other life stages was indeed found in waterfowl [55], it suggests an overall lower repeatability of CORT secreted during molt compared to other part of the life cycle.

External factors have been shown to have a strong influence on molt in birds, affecting timing of onset and rate of molt [57]. Our study suggests that feather CORT levels were more influenced by environmental conditions experienced by female eiders during the molting period than by individual quality.

The large inter-annual variability that we observed in eider feather CORT was not related to differences in breeding investment, timing of breeding or pre-breeding body condition prior to molting. This result contrasts with Bortolotti et al. [6] which clearly demonstrates a positive relationship between breeding investment and CORT in feathers grown in late summer of the same year in captive red-legged partridges (*Alectoris rufa*). It is surprising that this relationship was not observed in female common eiders, since they lose up to 40% of their mass during incubation [33]. We expected that such an important energetic demand would affect an individual's physiology up to and during the molting period. Based on different metrics of breeding investment, our results nonetheless indicate that the previous state of an individual (e.g., reproduction and associated fasting during incubation) did not lead to stress-associated carry-over effects during the molting period (e.g., through increased stress responses or energetic demands). Foraging conditions experienced during the summer and early fall could potentially affect eider condition and thus their CORT levels during molt. The lack of individual repeatability and the large inter-annual variability that we observed in eiders CORT feathers suggests an important role for environmental conditions affecting stress experienced during molt.

The positive association between August and September temperatures and feather CORT may reflect a causal link between weather and stress during or just prior to molting when females rebuild their energy fuels. A direct link may occur through increased metabolic activity associated with thermoregulation. However, Jensen et al. [58] reported similar metabolic rates (although not measured during molt) in captive common eiders at 1.5 or 16–25°C which gives little support for a direct link between temperature and CORT in feathers. Temperature can likely affect stress during molt indirectly, through food quality and quantity; for instance, the profitability (flesh-shell ratio) of bivalves, a key food resource for eiders [34], increases when temperature is cold [59]. Petersen et al. [60] have also shown that filtration rate of arctic bivalves starts to decrease when water temperature was >6 degrees.

Our understanding of the importance of environmental effects on feather CORT in common eiders is based on data spanning five years. Establishing the mechanisms that affect energetic costs and stress responses during molting will require further studies either using longer-term data or experimental manipulation. Our study nonetheless provides evidence that CORT measured in feathers likely reflects environmental conditions [8] and has the potential to be a valuable tool to study carry-over effects [61], [62].
